# Differences between inflammatory cells infiltrated into tunica intima, media, and adventitia of ascending aortic aneurysms within diabetic and hypertensive patients

**DOI:** 10.17305/bb.2022.8565

**Published:** 2023-08-01

**Authors:** Aleksandra Milutinović, Marko Zivin, Ruda Zorc-Pleskovič

**Affiliations:** 1 Institute of Histology and Embryology, Faculty of Medicine, University of Ljubljana, Ljubljana, Slovenia; 2 International Center for Cardiovascular Diseases MC Medicor d.d., Izola, Slovenia; 3 Institute of Pathophysiology, Faculty of Medicine, University of Ljubljana, Ljubljana, Slovenia

**Keywords:** Type 2 diabetes (DM), arterial hypertension (AH), ascending aortic aneurysm, intima, media, adventitia, inflammation

## Abstract

The risk factors that are the most significant for the development of most cardiovascular diseases are arterial hypertension (AH), type 2 diabetes (DM), and inflammation. However, for the development of aortic aneurysms, DM is not one of them. Our study aimed to evaluate the difference between inflammatory infiltration in three individual layers of the ascending aortic aneurysm within diabetic and hypertensive patients. Forty-five patients aged 36–80 were divided into a group with diabetic patients without AH (group DM, *N* ═ 8) and hypertensive patients without DM (group AH, *N* ═ 37). For the histological analysis, aortic aneurysms were stained with hematoxylin eosin and Movat. We used immunochemical methods to detect pro- (M1), anti-inflammatory (M2) macrophages, T-helper, T-killer cells, B cells, and plasma cells. Statistical analysis was done by independent samples Kruskal–Wallis test adjusted by Bonferroni correction for multiple tests (*P* < 0.05). We found no difference in the volume density of collagen, elastin, vascular smooth muscle cells (VSMCs), and ground substance between groups. In the DM group, there were significantly fewer M2, T-helpers, and T-killers in the media than in the intima and the adventitia (*P* < 0.05). There were no significant differences in the number of M1, B, and plasma cells between all three vascular layers (*P* < 0.05). In the AH group, there were significantly fewer B and plasma cells, T-helper, T-killer cells, M1, and M2 in the media than in the intima and adventitia (*P* < 0.05). Our results conclude that the tunica media in the aneurismal wall of the AH group retained immunoprivileged. In contrast, in the DM group, all three layers were immunoprivileged.

## Introduction

Aortic aneurysms are defined as the permanent enlargement of the aortic lumen caused by the weakening of the vascular wall. Aortic aneurysms are often asymptomatic but can lead to rupture of the aortic wall, which is fatal in 80% of cases [1, 2].

Aneurysm development is a complex process involving an unbalanced relationship between the tension and strength of the aortic wall [1, 3] and inflammation [1, 4]. Numerous studies on human and experimental aneurysms have shown infiltration of inflammatory cells in the aneurysmal wall, including neutrophils, macrophages, T cells, B cells, dendritic, mast, and natural killer cells, and elevated levels of proteases and cytokines [5–8].

The aorta is the largest artery in the body. It belongs to the arteries of the elastic type. The structure of the normal wall of the aorta varies slightly depending on its section. Thus, the ascending part of the thoracic aorta has a thinner tunica intima and a thicker tunica media, with more elastic lamellae and collagen and a lower collagen-to-elastin ratio than the abdominal apart [9–11]. Aneurysms in the thoracic and abdominal part of the aorta also differ slightly according to the major pathological changes in the wall. For a long time, the prevailing opinion was that the formation of an abdominal aortic aneurysm is associated with inflammation and atherosclerosis, while the development of an aneurysm in the thoracic part is more degenerative, non-inflammatory in nature [12]. More recent studies have discovered that low-grade chronic inflammation is also involved in the development of aneurysms in the thoracic part of the aorta [13–15]. Moreover, inflammatory infiltration was found to be also quite similar in both locations. Among the inflammatory cells infiltrated in the aneurismal wall, in the abdominal and also thoracic part, T cells were found to be the most numerous, followed by macrophages, B cells, and plasma cells [8, 9, 14–17]. It was reported that the majority of the T cells population are T helper cells (Th) [8, 17, 18] which are involved in macrophage activation, direct destruction of the aortic wall, and promotion of vascular smooth muscle cells (VSMCs) apoptosis [8, 19]. Killer T cells (Tk) are rare in the aneurysm wall [17]. They also contribute to the development of aneurysms by promoting matrix metalloprotease activity and cellular apoptosis [18, 20].

Macrophages are one of the most important cells involved in the innate defense of the organism. The essential functions of macrophages are phagocytosis and antigen presentation [6]. In 2000, pro- (M1) and anti-inflammatory (M2) phenotypes of macrophages were discovered [21, 22]. Aortic aneurysm development was shown to be promoted by M1 macrophages and prevented by M2 macrophages [22].

They found that B cells are rare in the aneurysm wall. Nevertheless, B cells and proinflammatory immunoglobulins promote aneurysm development [23].

Risk factors for an aortic aneurysm are very similar to those involved in developing other cardiovascular diseases: hypertension, male gender, age, smoking, family history, and atherosclerosis, but not diabetes [1, 24]. Aortic aneurysms in the abdominal and thoracic part develop almost 30% less often in patients with diabetes type 2 (DM) than in non-diabetic patients [8, 25].

It is well known that DM and arterial hypertension (AH) are both associated with chronic, low-grade inflammation [26, 27]. Recently, our research group reported that the wall of an aortic aneurysm in diabetic patients was less infiltrated with B and plasma cells and M1 macrophages compared to inflammatory infiltration in aneurysm of hypertensives. The minor infiltration of B and M1 cells was found in the tunica intima, tunica media, and plasma cells were found in the tunica media and tunica adventitia [17].

We aimed to compare the infiltration of different types of inflammatory cells between tunica intima, tunica media, and tunica adventitia of ascending aortic aneurysms within DM and AH groups of patients.

## Materials and methods

### Patients

45 patients with ascending aortic aneurysms were enrolled in the study. The patients had either DM (group DM; *N* ═ 8) or AH (group AH; *N* ═ 37). The exclusion criteria were DM and AH, DM type 1, and genetic mutations [17].

### Tissue samples

Samples of ascending aortic aneurysms were taken during surgery ranging from 2 to 5 cm. Three samples were obtained from the bulge area of aneurysms, fixed in formalin, embedded in paraffin, and cut into 4.5-µm thick stepped serial sections on a microtome. The thickness of the step was 50 µm. Sections were stained with hematoxylin eosin (HE) and Movat pentachrome to detect collagen fibers, elastic fibers, VSMC, and ground substance [28]. For the detection of M1-macrophages (anti-CD68,1: 50, Dako, Glostrup, Denmark), M2-macrophages (anti-CD163 1:40, Cell Marque), Th cells (anti-CD4; 1:20, Cell Marque), Tk cells (anti-CD8 1:50, Dako, Glostrup, Denmark), B cells (anti-CD79a; 1:20, Dako Glostrup, Denmark), and plasma cells (anti-CD138,1: 30, Dako, Glostrup, Denmark) the immunohistochemical staining was used according to the manufacturer’s instructions [17].

### Image analysis

Image analysis was performed using a light microscope (Nikon Eclipse E 400), a camera (Nikon digital sight DS-M5), and the computer program NIS elements (version 3 – D). The area (mm^2^) of tunica intima, tunica media, and tunica adventitia was measured. The tunica adventitia was defined as connective tissue lying adjacent to the tunica media in a 500-µm wide band.

The number (*N*) of CD68, CD163 CD4, CD8, CD79a, and CD138 positive cells in intima, media, and adventitia was counted in the whole section at an objective magnification of 40× and expressed in N/mm^2^.

For stereological analysis with Weibel’s test system, Movat pentachrome staining was used. In the intima, media, and adventitia, the volume density (mm^3^/mm^3^) of elastic (black) and collagen fibers (yellow), VSMC (dark red), and ground substance (blue-green) were estimated as described previously [13, 21].

### Ethical statement

The study protocol was written and conducted by the ethical guidelines of the 1975 Declaration of Helsinki. Written consent was obtained from each patient included in the study. The National Medical Ethics Committee approved the study and all procedures (MEC 170/07/13, MEC 110/03/16).

### Statistical analysis

Analyses were performed using Statistical Package for Social Sciences SPSS 20 and Microsoft Excel 2010.

The average values of the measured parameters (collagen and elastic fibers, VSMC, and ground substance and the number of CD68, CD163, CD4, CD8, CD79a, and CD138 positive cells) in tunica intima, tunica media, and tunica adventitia within DM and AH groups were calculated and expressed as the average value ± SD.

The differences of measured parameters between tunica intima, tunica media, and tunica adventitia of aneurismal wall in the DM and AH groups were tested with Kruskal–Wallis for independent samples (Shapiro–Wilk normality test showed non-normal distribution) adjusted by Bonferroni correction for multiple tests (*P* < 0.05).

The statistical significance of the differences between the DM and AH groups was evaluated by Welch’s ANOVA test (*P* < 0.05) (unequally sized samples).

## Results

### Patients

The patients were divided into the DM (*N* ═ 8) and AH groups (*N* ═ 37). There were more men than women and more non-smokers and ex-smokers than smokers in both groups (Table 1). The groups did not differ significantly in age, total plasma cholesterol, low-density lipoprotein, high-density lipoprotein, triglycerides, creatinine, and urea (Table 1).

**Table 1:** Characteristics for all patients in the AH and DM group (Welch test, *P* < 0.05)

**Variable**	**DM group**	**AH group**	***P* value**
	*N* ═ 8	*N* ═ 37	
Age (years)	63.13 ± 12.61	57.37 ± 8.68	0.253
Sex	7 male, 1 female	31 male, 6 female	
Smoking	yes 0; no 8, stopped smoking 2	yes 6; no 31, stopped smoking 2	
Total cholesterol (mmol/L)	5.36 ± 1.11	5.03 ± 0.57	0.414
LDL (mmol/L)	3.93 ± 1.16	3.29 ± 0.71	0.171
HDL (mmol/L)	1.22 ± 0.40	1.38 ± 0.52	0.344
Triglyceride (mmol/L)	2.46 ± 1.00	1.72 ± 0.77	0.103
Creatinine (µmol/L)	80.83 ± 11.58	81.61 ± 10.96	0.883
Urea (mmol/L)	7.28 ± 1.66	6.76 ± 1.55	0.492

Note that there were no significant differences in measured parameters between groups. DM: Type 2 diabetes; AH: Arterial hypertension; LDL: Low-density lipoprotein; HDL: High-density lipoprotein.

### Histological analysis of the volume density of tissue components between tunica intima, tunica media, and tunica adventitia within the DM and AH groups

**Figure 1.**
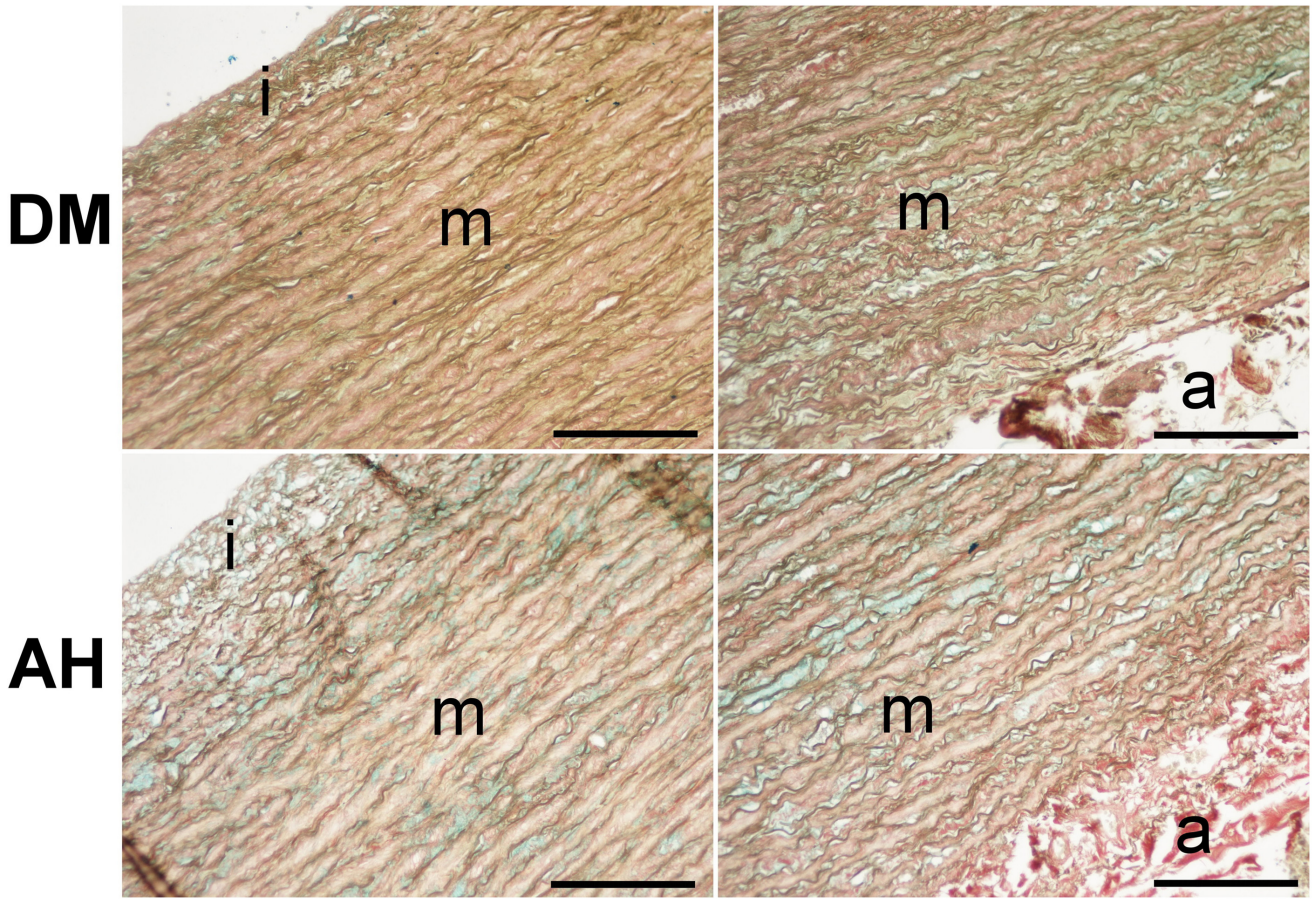
**Movat staining of ascending aortic aneurysm in the DM and AH group; tunica intima (i), tunica media (m), and tunica adventitia (a)**. Bar ═ 150 µm, objective magnification of 20×. DM: Type 2 diabetes; AH: Arterial hypertension.

The samples of ascending aortic aneurysms were stained with HE and Movat. The tunica adventitia of DM and AH groups contained the most collagen, a few elastic fibers, and ground substance, while the tunica media contained the most VSMC (Figure 1).

**Figure 2.**
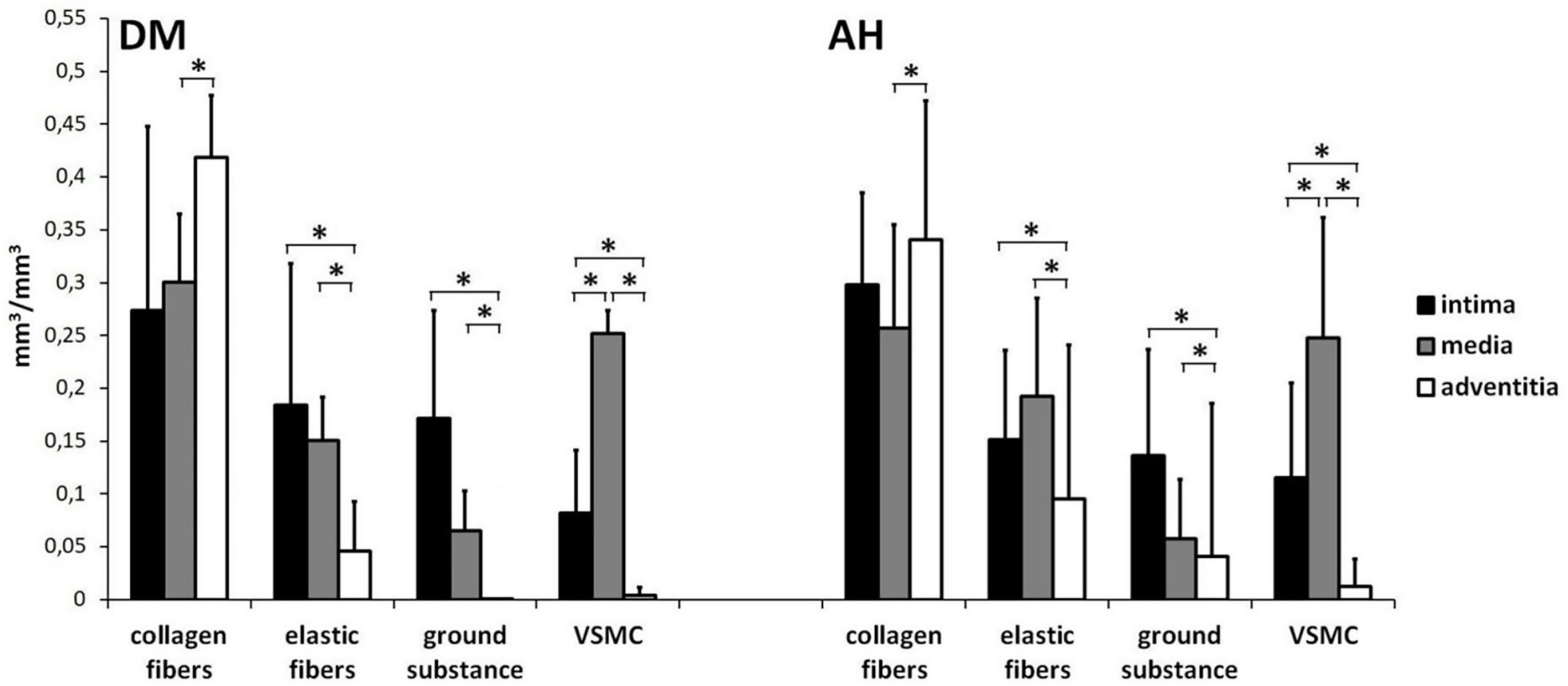
The volume density of collagen and elastic fibers, VSMC, and ground substance between intima, media, and adventitia within the DM and AH group (*significantly different, Kruskal–Wallis test, *P* < 0.05). Note that there were the same significant differences in the volume density of collagen and elastic fibers, ground substance, and VSMC between the tunica intima, tunica media, and tunica adventitia in both groups—lower volume density of VSMC in the tunica intima than in the tunica media, lower volume density of elastic fibers, ground substances, and VSMC in the tunica adventitia than in the tunica intima, and lower volume density of collagen and elastic fibers, ground substances, and VSMC in the tunica adventitia than in the tunica media. VSMC: Vascular smooth muscle cells; DM: Type 2 diabetes; AH: Arterial hypertension.

Stereological analysis showed equally significant differences in the volume density of collagen and elastic fibers, VSMC and ground substance, and VSMC between the tunica intima, tunica media, and tunica adventitia within the DM and AH groups. In both groups, we found significantly lower volume density of collagen and elastic fibers, ground substances, and VSMC in the tunica adventitia than in the tunica media, significantly lower volume density of elastic fibers, ground substances, and VSMC in the tunica adventitia than in the tunica intima, and significantly lower volume density of VSMC in tunica intima than in tunica media (Figures 1 and 2; Table 2).

**Table 2:** Average values of volume density of collagen and elastic fibers, ground substance, and VSMC in tunica intima, tunica media, and tunica adventitia within DM and AH group (addition to the Figure 1)

	**DM group**			**AH group**		
**mm^3^/mm^3^ ± SD**	**Tunica intima**	**Tunica media**	**Tunica adventitia**	**Tunica intima**	**Tunica media**	**Tunica adventitia**
Collagen fibers	0.27 ± 0.17	0.30 ± 0.06	0.42 ± 0.06	0.30 ± 0.09	0.26 ± 0.10	0.34 ± 0.13
Elastic fibers	0.18 ± 0.13	0.15 ± 0.04	0.05 ± 0.05	0.15 ± 0.08	0.19 ± 0.09	0.10 ± 0.15
Ground substance	0.17 ± 0.10	0.07 ± 0.04	0.00 ± 0.00	0.1 ± 0.10	0.06 ± 0.06	0.04 ± 0.15
VSMC	0.08 ± 0.06	0.25 ± 0.02	0.01 ± 0.01	0.12 ± 0.09	0.25 ± 0.11	0.01 ± 0.03

VSMC: Vascular smooth muscle cells; DM: Type 2 diabetes; AH: Arterial hypertension.

### Histological analysis of the volume density of tissue components between DM and AH group

We found no significant differences in the volume density of collagen and elastic fibers, VSMC, and ground substance between the DM and AH groups (Figure 1; Table 2).

### Analysis of M1 and M2 macrophages, Th, Tk, B, and plasma cells between tunica intima, tunica media, and tunica adventitia within AH and DM group

#### DM group

**Figure 3.**
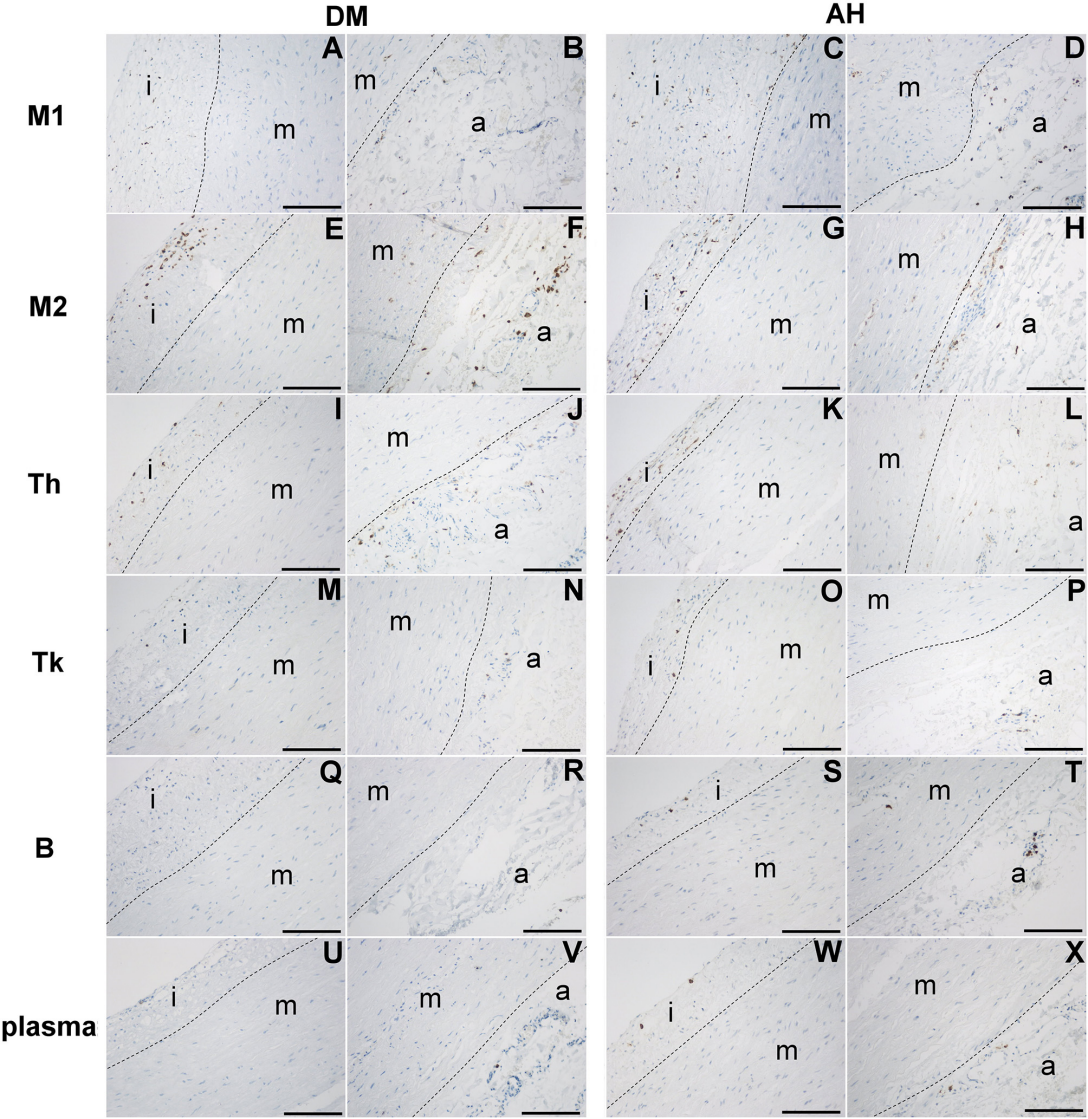
**M1 (anti-CD68) and M2 macrophages (anti-CD163), Th (anti-CD4), Tk (anti-CD8), B (anti-CD79A), and plasma cells (anti-CD138) in (i) tunica intima, (m) tunica media, and (a) tunica adventitia in the DM (A, B, E, F, I, J, M, N, Q, R, U, V) and AH (C, D, G, H, K, L, O, P, S, T, W, X) groups.** Bar ═ 150 µm, objective magnification of 20×. A dashed line shows the boundaries between intima, media, and adventitia. DM: Type 2 diabetes; AH: Arterial hypertension; Th: T helper cells; Tk: Killer T cells.

**Figure 4.**
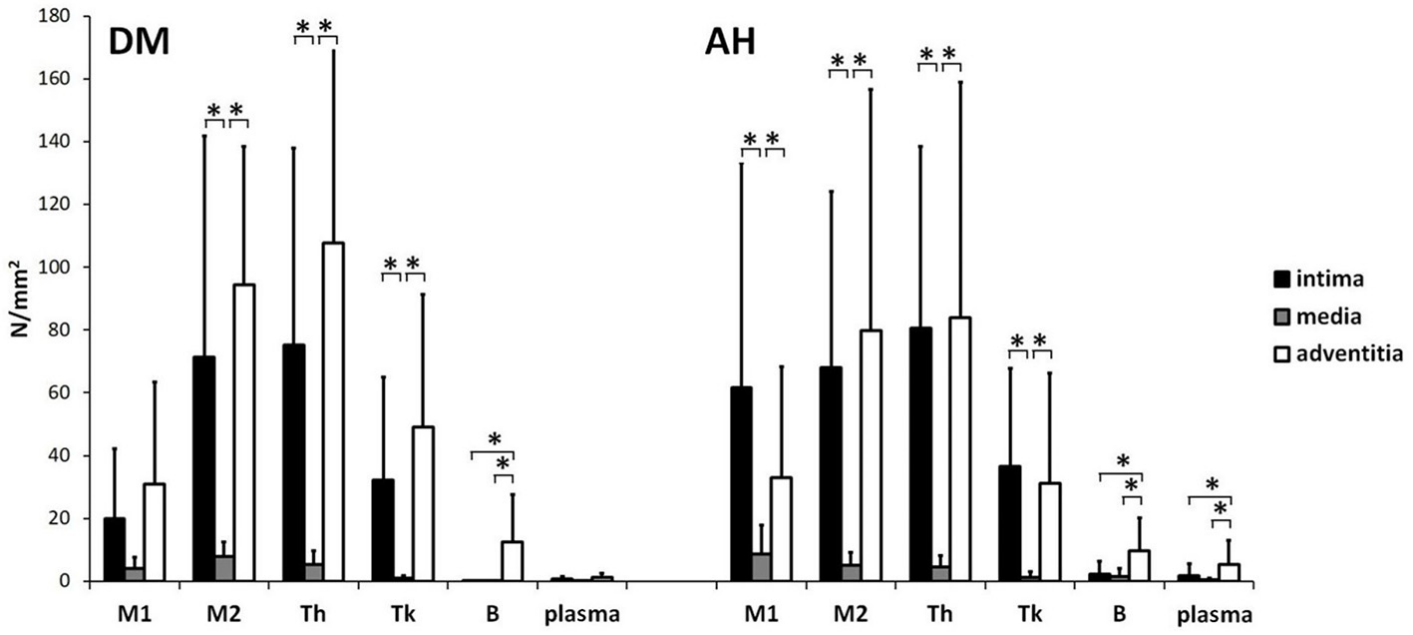
The number (N/mm^2^) of M1 (anti-CD68) and M2 macrophages (anti-CD163), Th (anti-CD4), Tk (anti-CD8), B (anti-CD79A), and plasma cells (anti-CD138) between tunica intima, tunica media, and tunica adventitia within the DM and AH group (*significantly different, Kruskal–Wallis test, *P* < 0.05). Note that in the DM group, there were no differences in the number of M1 and plasma cells between layers. However, in the AH group, there were fewer M1 cells in media compared to intima and adventitia and fewer plasma cells in intima and media than in adventitia. Between the tunica intima, tunica media, and tunica adventitia, there were the same significant differences in the number of M2, Th, Tk, and B cells in both groups. DM: Type 2 diabetes; AH: Arterial hypertension; Th: T helper cells; Tk: Killer T cells.

We found no significant differences in the number of plasma cells and M1 macrophages between tunica intima, tunica media, and tunica adventitia (Figures 3 (A, B, U, V) and 4; Table 3). There were fewer M2 macrophages Th and Tk cells in the tunica media than in the tunica intima and tunica adventitia. However, we found no differences in the number of M2, Th, and Tk cells between tunica intima and tunica adventitia (Figures 3 (E, F, I, J, M, N) and 4; Table 3). There were more B cells in the tunica adventitia than in the tunica intima and tunica media. Tunica intima and media did not differ in the number of B cells (Figures 3 (Q, R) and 4; Table 3).

**Table 3:** Average values of inflammatory cells in tunica intima, tunica media, and tunica adventitia within the DM and AH group (addition to Figures 3–5)

	**DM group**			**AH group**		
**N/mm^2^ ± SD**	**Tunica intima**	**Tunica media**	**Tunica adventitia**	**Tunica intima**	**Tunica media**	**Tunica adventitia**
M1 macrophages	19.9 ± 22.18	4.05 ± 3.49	30.80 ± 32.72	61.70 ± 71.63	8.52 ± 9.24	32.84 ± 35.40
M2 macrophages	71.37 ± 70.48	7.85 ± 4.66	94.54 ± 43.97	68.14 ± 56.00	5.12 ± 4.03	79.81 ± 76.97
Th cells	75.32 ± 62.58	5.41 ± 4.24	107.73 ± 62.38	80.62 ± 57.79	4.55 ± 3.65	83.84 ± 75.11
Tk cells	32.28 ± 32.80	0.92 ± 0.80	49.05 ± 42.42	36.47 ± 31.23	1.23 ± 1.70	31.16 ± 35.07
B cells	0.09 ± 0.15	0.05 ± 0.09	12.41 ± 15.18	2.26 ± 4.15	1.45 ± 2.4	9.62 ± 10.41
Plasma cells	0.65 ± 0.71	0.02 ± 0.04	1.14 ± 1.43	1.71 ± 3.80	0.30 ± 0.53	5.29 ± 7.76

DM: Type 2 diabetes; AH: Arterial hypertension; Th: T helper cells; Tk: Killer T cells.

#### AH group

There were fewer M1 and M2 macrophages, Th, and Tk cells in the tunica media than in the tunica intima and tunica adventitia. No significant differences were found between tunica intima and tunica adventitia in the number of M1, M2, Th, and Tk cells (Figures 3 (C, D, G, H, K, L, O, P) and 4; Table 3).

A higher number of B and plasma cells were found in tunica adventitia than in the tunica intima and tunica media. There was no difference in the number of B and plasma cells between the tunica media and the tunica intima (Figures 3 (S, T, W, X) and 4; Table 3).

### Comparison of inflammatory cells in tunica intima, tunica media, and tunica adventitia between the AH and DM group

**Figure 5.**
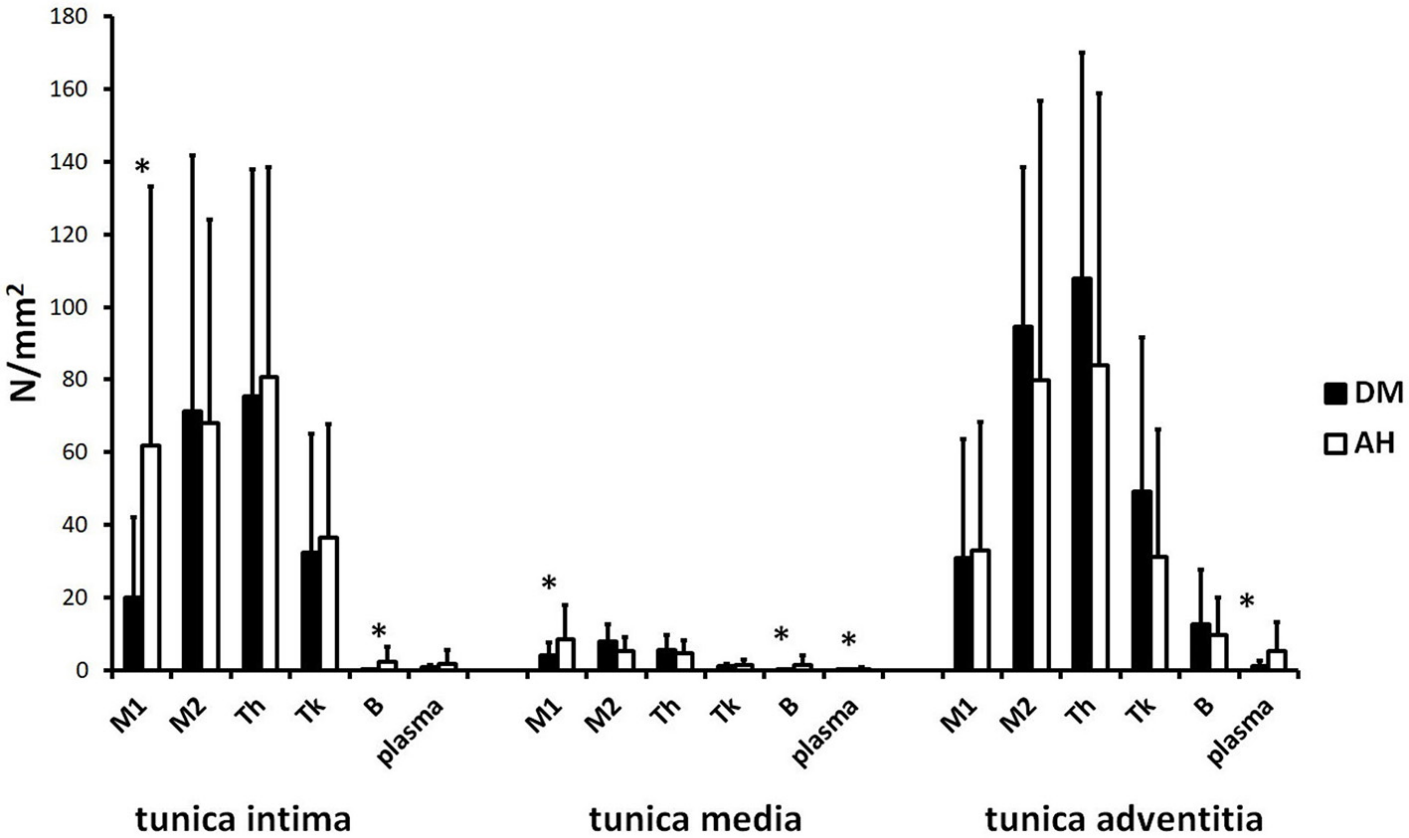
Analysis of inflammatory cells in the tunica intima, tunica media, and tunica adventitia of the ascending aortic aneurysm between the DM and AH group (*significantly different, Welch test, *P* < 0.05). In the DM group, there were significantly fewer M1 and B cells in the tunica intima and media and fewer plasma cells in media and adventitia than in the AH group. DM: Type 2 diabetes; AH: Arterial hypertension; Th: T helper cells; Tk: Killer T cells.

We found significantly lower infiltration of B and M1 cells in tunica intima and tunica media and fewer plasma cells in tunica media and tunica adventitia in the DM group than in the AH group. There were no significant differences in other inflammatory cell subtypes between both groups (Figures 3 and 5; Table 3).

## Discussion

In the present study, we compared the intensity of M1, M2, Th, Tk, B, and plasma cell infiltration between tunica intima, media, and adventitia of the aneurismal wall within a diabetic non-hypertensive and non-diabetic hypertensive group of patients. In the AH group, we found fewer M1, M2, Th, and Tk cells in the tunica media compared to intima and adventitia and fewer B and plasma cells in intima and media compared to the adventitia. In the DM group, we found fewer M2, Th, and Tk cells in the tunica media compared to the tunica intima and tunica adventitia and fewer B cells in intima and media compared to the adventitia. However, there were no significant differences in M1 and plasma cells among all three layers of the aneurysm wall in the DM group. Macrophages and T cells might enter into the vessel wall from the luminal part and surrounding tissue. However, most B and plasma cells primarily come from surrounding tissue.

The role of macrophages is very important for the innate immune response. They are phagocytes, antigen-presenting cells, and cytokine-secreting modulators of inflammation, immune defense, metabolism, tissue reconstruction, and homeostasis [6]. Macrophages originate either from embryonic precursors or from monocytes. Most macrophages found in the wall of an aortic aneurysm are derived from monocytes [8, 29]. In response to inflammatory stimuli, blood monocytes are recruited to the vascular tissue and differentiate into M1 or M2 macrophages. They have very different roles in the development of an aortic aneurysm. M1, as proinflammatory macrophages, aggravates local inflammation, dilatation, and remodeling of the aortic wall. M2 macrophages with anti-inflammatory function promote collagen deposition, cell recruitment, and angiogenesis in the aneurismal wall [8, 30]. In our study of diabetics and hypertensives, the stereological analysis showed that the tunica intima and tunica adventitia collagen fibers predominated in the connective tissue. In the tunica media, in addition to collagen fibers, VSMCs were the most abundant. We found no differences between DM and AH groups.

It was reported that in the abdominal aortic aneurism, M1 macrophages predominate in the tunica adventitia and M2 macrophages in an intramural thrombus. However, they did not find any macrophages in tunica media [31] because it retained its immunoprivilege [32].

In our current and recently published study [17], we observed M1 and M2 macrophages and Th, Tk, B, and plasma cells in all three wall layers in ascending thoracic aorta aneurysms in hypertensive and diabetic patients.

It is known that in inflammatory arterial diseases, the tunica media is less infiltrated by inflammatory cells than the tunica intima and tunica adventitia because passive mechanical and active biological mechanisms protect it—tunica media is immunoprivileged [32]. Mechanical passive protection of the tunica media includes elastic laminae or fenestrated elastic membranes, which represent a barrier to the passage of inflammatory cells and macromolecules, and the absence of blood and lymph vessels that prevent inflammatory responses. The tunica media is usually avascular, unless it consists of more than 29 laminas or the tunica intima is more thick than 0.35–0.5 mm [33]. The aorta is a vessel with a thick wall. For sufficient oxygenation, there are also blood vessels (vasa vasorum) in the outer part of the tunica media. Therefore, the outer part of the tunica media has poorer mechanical immunoprivilege as blood vessels are the pathways by which inflammatory cells get deeper into the tunica media [32, 33]. VSMCs play a major role in the active biological protection of the tunica media of the vascular wall. They secrete substances, such as the immunomodulatory enzyme indolamine 2,3-dioxygenase (IDO) and transforming growth factor beta (TGFβ) that inhibit the activation of macrophages and T cells. Consequently, they infiltrate only the tunica intima and tunica adventitia [32, 34]. In addition to VSMC, the absence of B cells also plays an essential role in immunoprivilege. An experimental model for an aortic aneurysm showed that mice without B cells had more IDO in the aortic wall, more regulatory T cells, and fewer proinflammatory cytokines than mice with B cells [35].

Leukocytes from patients with diabetes have increased expression of genes associated with inflammation [36]. It was reported that advanced glycation end products induce macrophages to differentiate into M1 macrophages and restrain M2 macrophages differentiation in MIN6 mice β cells line. They enhance M1 macrophages to excrete proinflammatory cytokines, such as IL1β, IL6, and TNFα [37]. Considering the effects of ACEs, we would predict that there would be a higher number of M1 cells in the DM group than in the AH group. However, in our study, we found fewer M1 and B cells in the tunica intima and tunica media of the DM group than in the AH group. We found fewer M2, Th, and Tk cells within the DM group in the tunica media than in the tunica intima and tunica adventitia. The number of M1 and plasma cells was the same in all three layers.

Within the AH group, we found fewer M2, Th, Tk, and M1 cells in the tunica media than in the tunica intima and tunica adventitia. There were fewer B cells in intima and media compared to the tunica adventitia in the DM and AH groups. The same number of B cells was found in the tunica intima and tunica media. Fewer B cells in the tunica intima and tunica media of the DM group in our study could result in a larger amount of IDO and fewer proinflammatory cytokines [35] that might inhibit the activation of macrophages which is thought to be caused by advanced glycation end products [37].

Recently, it was reported that macrophages from diabetic patients exhibit higher metabolic activity. They treated the culture of healthy primary human monocyte-derived macrophages with the serum of diabetic and non-diabetic patients with aortic aneurysms. They found that macrophages treated with the serum of diabetic patients increased fatty acid oxidation and glycolysis, which leads to higher extracellular acidification. Further analysis showed that macrophage metabolism changed. The inflammatory state was tested and found no differences in pro-fibrotic and anti-inflammatory cytokine TGFβ, a slight increase in the marker for M2 cells, a slight increase in cytokine with pro-inflammatory properties IL1β, an increase in anti-inflammatory cytokine IL10, and decrease in pro-inflammatory cytokine TNFα. They suggested that changes in macrophage metabolism caused by diabetic serum lead to reduced risk for the development of aortic aneurysm [38]. The finding that the metabolism of inflammatory cells is changed could be the reason that in our study, the amount of collagen and elastic fibers of VSMC and ground substance in the AH and DM groups is the same. However, in the DM group, there is less inflammatory infiltration in all three layers of the aneurysm wall.

It is known that both diabetes and AH are associated with low-grade, chronic inflammation [26, 27]. AH increases the risk of thoracic aortic aneurysm formation [39], while diabetes decreases it [40]. Glycation changes the metabolism of macrophages in the direction of anti-inflammatory state (M2), which stimulates the formation of collagen fibers, strengthens the vascular wall, and reduces the risk of abdominal aneurysm formation [38, 41, 42]. In our study, we had a group of patients who had diabetes and an aneurysm but did not have hypertension. Patients with diabetes, without hypertension and thoracic aortic aneurysm, are rare [17, 43, 44], so our DM group was very small (*N* ═ 8), which is the major limitation of this study. The aneurysm wall did not differ in the volume density of collagen, elastin, ground substance, and VSMC in the DM and AH groups in the current study. We hypothesize that these patients might have a different metabolism and/or body composition and/or effects of glycation and/or infiltration of inflammatory cells and their products and consequently a different course of aneurysm formation than the majority of diabetics who do not develop an aneurysm. Our study focused on inflammatory infiltration into the vessel wall. Within the AH group, we found fewer M1, M2, Th, and Tk M1 cells in the tunica media than in the tunica intima and tunica adventitia, indicating that the media retained immunoprivileged. Within the DM group, we found fewer M2, Th, and Tk cells in the tunica media than in the tunica intima and tunica adventitia. The number of M1 and plasma cells was the same in all three layers. We found fewer M1 and B cells in the intima and media and fewer plasma cells in the media and adventitia of the DM group than in the AH group. Since M1 and plasma cells with antibody secretion increase the inflammation, we could speculate that in the DM group, all three layers of the aneurysm wall are immunoprivileged. Further investigation of the mechanisms of aneurysm formation in these rare patients could contribute to the understanding of aneurysm formation as well as the effects of elevated plasma sugar and subsequent possible treatment in clinical practice.

## Conclusion

We conclude that tunica media of the wall of an aortic aneurysm in hypertensive patients retained immunoprivileged. In contrast, diabetic non-hypertensive patients have immunoprivileged all three layers of the aneurismal wall.
